# Intratumoral heterogeneity of intermediate CLDN18.2 expression in gastric cancer

**DOI:** 10.1007/s10147-026-03118-8

**Published:** 2026-07-07

**Authors:** Yusuke Kawanaka, Chiaki Inagaki, Masaki Okura, Seiichiro Mitani, Naoki Shiraishi, Kazuko Sakai, Kazuto Nishio, Yutaka Kimura, Yasutaka Chiba, Tomoko Wakasa, Hisato Kawakami, Hidetoshi Hayashi

**Affiliations:** 1 Department of Medical Oncology, Kindai University Faculty of Medicine, 1-14-1 Minami-ku, Sakai, Osaka 590-0197 Japan; 2 Genome Medical Center, Kindai University Hospital, Sakai, Osaka Japan; 3 Department of Genome Biology, Kindai University Faculty of Medicine, Sakai, Osaka Japan; 4 Department of Surgery, Kindai University Nara Hospital, Ikoma, Nara Japan; 5 Clinical Research Center, Kindai University Hospital, Sakai, Osaka Japan; 6 Department of Diagnostic Pathology, Kindai University Nara Hospital, Ikoma, Nara Japan; 7 Department of Clinical Oncology, Tohoku University Graduate School of Medicine, Sendai, Miyagi Japan

**Keywords:** Gastric cancer, CLDN18.2, Immunohistochemistry, Heterogeneity, Concordance

## Abstract

**Background:**

CLDN18.2-targeted therapies show promise in HER2-negative, CLDN18.2-positive advanced gastric cancer (GC); however, cutoffs defining CLDN18.2 positivity vary across studies. Tumors with high expression (moderate to strong membranous expression in ≥ 75% of tumor cells) generally exhibit intratumoral homogeneity and a uniform staining pattern, but intratumoral heterogeneity and staining pattern of CLDN18.2 at lower expression thresholds remains unclear. This study investigated intratumoral heterogeneity of CLDN18.2 by comparing inter-block concordance of expression levels and staining patterns in GC.

**Methods:**

Eighty-six resected GC specimens were retrospectively analyzed using CLDN18.2 immunohistochemistry on two tissue blocks per tumor. CLDN18.2 expression was categorized as high (≥ 75%), intermediate (< 75% and ≥ 40%), or negative (< 40%), and staining patterns as uniform or variable. Inter-block concordance of expression levels and staining patterns and their influence on concordance and outcomes were assessed.

**Results:**

Overall inter-block concordance of expression levels was high (77%, κ = 0.89). Discordance was substantially higher in intermediate expression tumors (63.5%) than in high (15.5%) or negative (19%) expression tumors. Uniform staining was observed exclusively in high expression tumors, whereas all intermediate expression tumors displayed variable staining (100%). Among high expression tumors, inter-block concordance was substantially higher with uniform staining (98.0%) than with variable staining (71.5%). CLDN18.2 expression and staining pattern heterogeneity were not associated with worse survival.

**Conclusion:**

Significant intratumoral heterogeneity of CLDN18.2 expression was observed in GC, particularly in intermediate expression tumors; however, this heterogeneity does not affect survival. These results highlight the importance of standardized assessment methods when applying lower thresholds for CLDN18.2-targeted therapies.

**Supplementary Information:**

The online version contains supplementary material available at 10.1007/s10147-026-03118-8.

## Introduction

Gastric cancer (GC) remains one of the most lethal malignancies, ranking sixth in incidence and third in mortality worldwide despite a gradual global decline [1]. Perioperative chemotherapy and resection are standard treatments for resectable GC, whereas chemotherapy is the primary treatment for advanced unresectable and/or metastatic GC [2]. Recent advances in biomarker-driven treatment strategies necessitate assessing human epidermal growth factor receptor type 2 (HER2), mismatch repair/microsatellite instability-high, programmed death ligand 1 combined positive score, and Claudin-18 isoform 2 (CLDN18.2) status to guide first-line treatment selection in advanced GC [2].

CLDN18.2 is a variant of claudin18, a transmembrane protein that forms part of the tight junctions between normal epithelial cells of the gastric mucosa [3]. In normal mucosa, CLDN18.2 is largely inaccessible to antibodies; however, malignant transformation in GC leads to a loss of cell polarity [4], exposing the CLDN18.2 epitope and enhancing its accessibility to antibody. Zolbetuximab, a mouse chimeric monoclonal antibody with a human IgG1 constant region that specifically binds to CLDN18.2, has shown efficacy in GC [5–7]. The SPOTLIGHT and GLOW phase III trials demonstrated that zolbetuximab combined with chemotherapy improved overall survival (OS) and progression-free survival in HER2-negative, CLDN18.2-positive, treatment-naïve GC compared with chemotherapy alone [6, 7]. In these trials, CLDN18.2-positive advanced GC was defined as tumors showing moderate (2 +) to strong (3 +) membranous CLDN18.2 immunohistochemistry (IHC) expression in at least 75% of tumor cells (high expression). Meanwhile, several investigational agents beyond zolbetuximab, including antibody–drug conjugates [8, 9] and CAR T-cell therapies [10], are under active development. These investigational therapies use lower thresholds for CLDN18.2 positivity, such as intermediate to strong membranous expression in ≥ 40% of tumor cells (intermediate expression) for target population selection.

GC is characterized by substantial intratumoral heterogeneity [11], particularly in the spatial expression of key molecular markers [12–17], posing substantial challenges in biomarker testing accuracy [12]. CLDN18.2-high (≥ 75% of tumor cells) tumors generally exhibit intratumoral homogeneous expression within the primary tumor [18], although some studies report heterogeneity [19]. Additionally, intratumoral heterogeneity in staining intensity (staining pattern) has been indicated in approximately 31.0–38.5% of cases [20, 21]. In contrast, the extent of intratumoral heterogeneity in expression and staining patterns among intermediate CLDN18.2 expression tumors (≥ 40% to < 75% of tumor cells) remains unclear [22].

Given the rapid development of CLDN18.2-targeted therapies, understanding intratumoral heterogeneity across different expression levels is crucial [22]. To address these points, we assessed intratumoral heterogeneity of CLDN18.2 expression and staining patterns using two formalin-fixed paraffin-embedded (FFPE) blocks from resected GC specimens, and examined the association between staining patterns and inter-block variability.

## Patients and methods

### Patients

This retrospective study included patients who underwent GC resection at Kindai University Nara Hospital between January 2015 and December 2021, excluding those who received preoperative chemotherapy. Clinical data, including patient age, sex, primary tumor location, histological classification (Lauren classification), pathological stage, HER2 status, and survival outcomes, were obtained from electronic medical records. The study was approved by the Institutional Review Board of Kindai University Faculty of Medicine (approval no. R05-115) and conducted in accordance with the Declaration of Helsinki. Due to the retrospective design of the study, the requirement for written informed consent was waived and an opt-out approach was adopted.

### Tissue preparation and immunohistochemical staining

Formalin fixation was performed in accordance with the Practical Guidelines of the Japanese Society of Pathology [23]. Surgical specimens were stored at 4 °C and fixed within 1 h after resection using 10% neutral buffered formalin (Fujifilm Wako Pure Chemical Corporation, Tokyo, Japan) for 6–48 h [23, 24]. Tissue sectioning was performed according to the Japanese Classification of Gastric Carcinoma [25]. Specifically, an initial section was taken along the lesser curvature as a reference line, with the deepest invasion area sectioned parallel to it. Subsequently, additional sections were obtained along multiple cut lines based on tumor size to evaluate surgical margins. All hematoxylin and eosin-stained slides from blocks were reviewed, and two representative sections, “block A” and “block B,” were selected to include the most well- and poorly differentiated histological areas. IHC for CLDN18.2 was performed using a standard protocol with a companion diagnostic antibody as previously described [21]. Briefly, thinly sliced FFPE blocks were serially deparaffinized and rehydrated using xylene and ethanol. Endogenous peroxidase was blocked via incubation in 3% H_2_O_2_ for 10 min, followed by heat-induced antigen retrieval. IHC staining was performed using a claudin18.2 antibody (clone 43-14A, Ventana Medical Systems, Tucson, AZ) with an autostainer (Benchmark XT, Ventana Medical Systems, Tucson, AZ) and an OptiView DAB Detection Kit (Ventana Medical Systems, Tucson, AZ).

### Evaluation of CLDN18.2 IHC staining

CLDN18.2 IHC staining intensity was evaluated based on the magnification rule of the United States and Canadian Academy of Pathology [26]. Specifically, a score of 3 + indicated strong brown membranous staining with a chicken-wire distribution visible at low magnification (× 2.5– × 5 objective/ × 25– × 50 magnification). A score of 2 + was assigned when membranous staining was visible at × 10 objective and confirmed at × 20. A score of 1 + indicated staining visible at × 20, requiring × 40 for confirmation, and interpreted as negative. Based on the proportion of tumor cells showing moderate (2 +) or strong (3 +) membranous CLDN18.2 staining, specimens were classified as high (≥ 75% of tumor cells), intermediate (≥ 40% to < 75%), or negative expression (< 40%, Online Resource 1). Cases with high or intermediate expression in both blocks were further classified as uniform or variable based on staining intensity patterns of CLDN18.2 [27] (Online Resource 2). The IHC evaluation was conducted by two observers (researcher Y.K and board-certified pathologist T.W). Interobserver agreement in the immunohistochemical assessment between two observers was evaluated using weighted κ statistics.

### Statistical analyses

The frequency distribution of CLDN18.2 expression levels (high, intermediate, and negative) was calculated independently for blocks A and B. In addition, each block was assessed for staining patterns (uniform, variable) across high or intermediate expression tumors. Inter-block concordance was quantified using weighted κ statistics. Concordance rates were averaged by comparing the assigned expression levels and staining patterns between the two blocks.

**Fig. 1:**
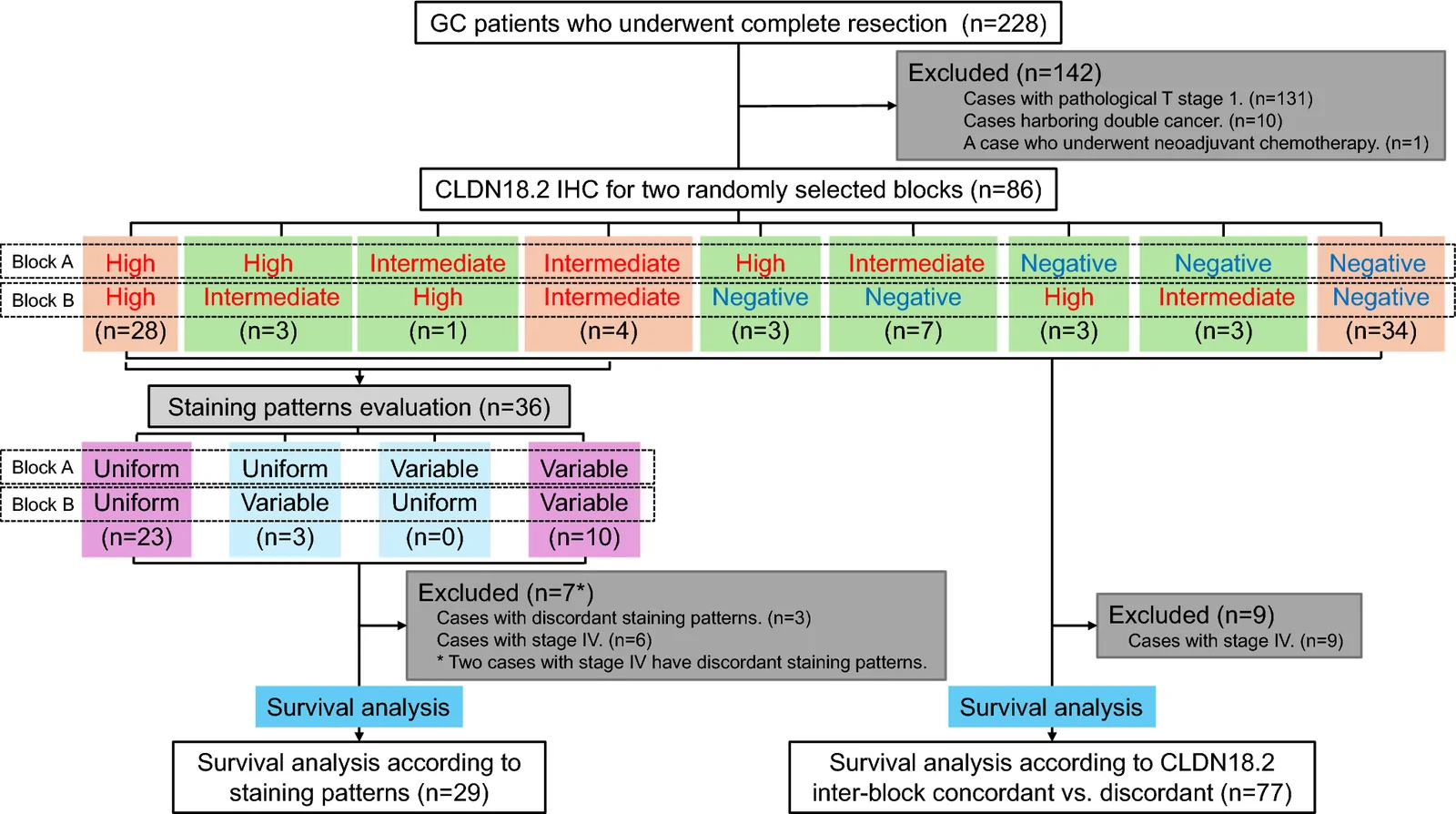
CONSORT diagram of the study. GC, gastric cancer; IHC, immunohistochemistry; High, high expression; intermediate, intermediate expression; Negative, negative expression

Survival analyses excluded patients with stage IV disease (Fig. 1). Recurrence-free survival (RFS) was defined as the interval between the date of complete resection (R0/R1) and the date of either disease recurrence or death from any cause, whichever occurred first. OS was defined as the time from complete resection to death from any cause. Kaplan–Meier survival curves were generated for RFS and OS, with *p*-values calculated using the log-rank test.

All statistical analyses were performed using JMP^®^ Pro 18 software (JMP Statistical Discovery LLC, Cary, NC, USA) and GraphPad Prism version 10.2.2 for Windows (GraphPad Software, Boston, Massachusetts USA, www.graphpad.com).

## Results

### Patient characteristics

In total, 86 patients were included in this study. Clinicopathological characteristics are summarized in Table 1. The median age was 77 years (range 36–94 years) and 73% (63/86) were male. The primary tumor was predominantly located in the lower stomach (58%, n = 50). Tumors were histologically classified into intestinal (45%, n = 39), diffuse (27%, n = 23), mixed (15%, n = 13), and indeterminate (13%, n = 11) types.

**Table 1:** Patient characteristics of overall study population

	Total
Characteristic	n = 86
Age, years	
Median (range)	77 (36–94)
Sex, n (%)	
Male	63 (73)
Female	23 (27)
Localization, n (%)	
Upper	10 (12)
Middle	26 (30)
Lower	50 (58)
TNM staging, n (%)	
I	16 (19)
II	36 (42)
III	25 (29)
IV	9 (10)
T stage, n (%)	
2	28 (33)
3	39 (45)
4	19 (22)
N stage, n (%)	
0	33 (38)
1	18 (21)
2	14 (16)
3	21 (24)
Histology, n (%)	
Intestinal	39 (45)
Diffuse	23 (27)
Mixed	13 (15)
Indeterminate	11(13)
Heavy alcohol consumption, n (%)*^1^	
Yes	2 (2)
No	22 (26)
Unknown	62 (72)
Smoking, n (%)	
Yes	11 (13)
No	11 (13)
Unknown	64 (74)
HER2 expression, n (%)	
Positive	9 (10)
Negative	50 (58)
Unknown	27 (31)
Complete resection, n (%)	
R0/1	77 (90)
R2	9 (10)
Adjuvant therapy *^2^	
Yes, Regimen, n (%)	35 (57)
TS-1	28 (46)
XELOX	2 (3)
SOX	3 (5)
DS	2 (3)
No, n (%)	26 (43)
Recurrence *^3^	
Yes, Sites, n (%)	27 (35)
Peritoneum	13 (48)
Liver	5 (19)
Lymph node	11 (41)
Lung	3 (11)
Local recurrence	4 (15)
Ascites	14 (52)
No, n (%)	50 (65)

HER2, human epidermal growth factor receptor 2; TS-1, tegafur, gimestat and otastat potassium combined drug; XELOX, combination of capecitabine and oxaliplatin; SOX, combination of tegafur, gimestat and otastat potassium combined drug and oxaliplatin; DS, combination of cisplatin and tegafur, gimestat and otastat potassium combined drug

*^1^Heavy alcohol consumption was defined as consuming ≥ 40 g of pure alcohol per day

*^2^Patients with pathological stage II or III gastric cancer were included

*^3^Patients with pathological stage I–III gastric cancer were included

### Categorization of CLDN18.2 expression levels and inter-block concordance

CLDN18.2 expression levels in blocks A and B were first categorized. In block A, 40% of tumors were high (n = 34), 14% intermediate (n = 12), and 46% negative (n = 40, Table 2). In block B, 37% were high (n = 32), 12% intermediate (n = 10), and 51% negative (n = 44). Analysis of interobserver agreement in IHC assessment showed high concordance (κ = 0.97 for block A and 0.98 for block B, Online Resource 3). To evaluate whether specimen storage duration influenced CLDN18.2 staining results, cases were stratified into two chronological periods (2015–2017 and 2018–2021). The frequencies of high, intermediate, and negative CLDN18.2 expression were comparable between the two periods in both block A (42%, 14%, 44% vs. 36%, 14%, 50%) and block B (38%, 10%, 52% vs. 33%, 14%, 53%) respectively. These findings suggest that storage duration did not substantially affect CLDN18.2 immunohistochemical results in this cohort (Online Resource 4). Clinicopathological characteristics were comparable across expression levels (Online Resource 5; Online Resource 6). In both blocks, intestinal type tumors were more frequently observed than diffuse type tumors in the high expression group (Block A: 55% [19/34] vs. 24% [8/34], Block B: 47% [15/32] vs. 31% [10/32]).

**Table 2:** Summary of CLDN18.2 immunohistochemistry expression levels in block A and block B

		Block B			
		High expression	Intermediate expression	Negative	Sum
Block A	High expression	**28**	*3*	*3*	34
	Intermediate expression	*1*	**4**	*7*	12
	Negative	*3*	*3*	**34**	40
	Sum	32	10	44	86

Bold highlights indicate expression levels concordant inter-block

Italic highlights indicate expression levels discordant inter-block

Weighted κ value = 0.89

**Fig. 2:**
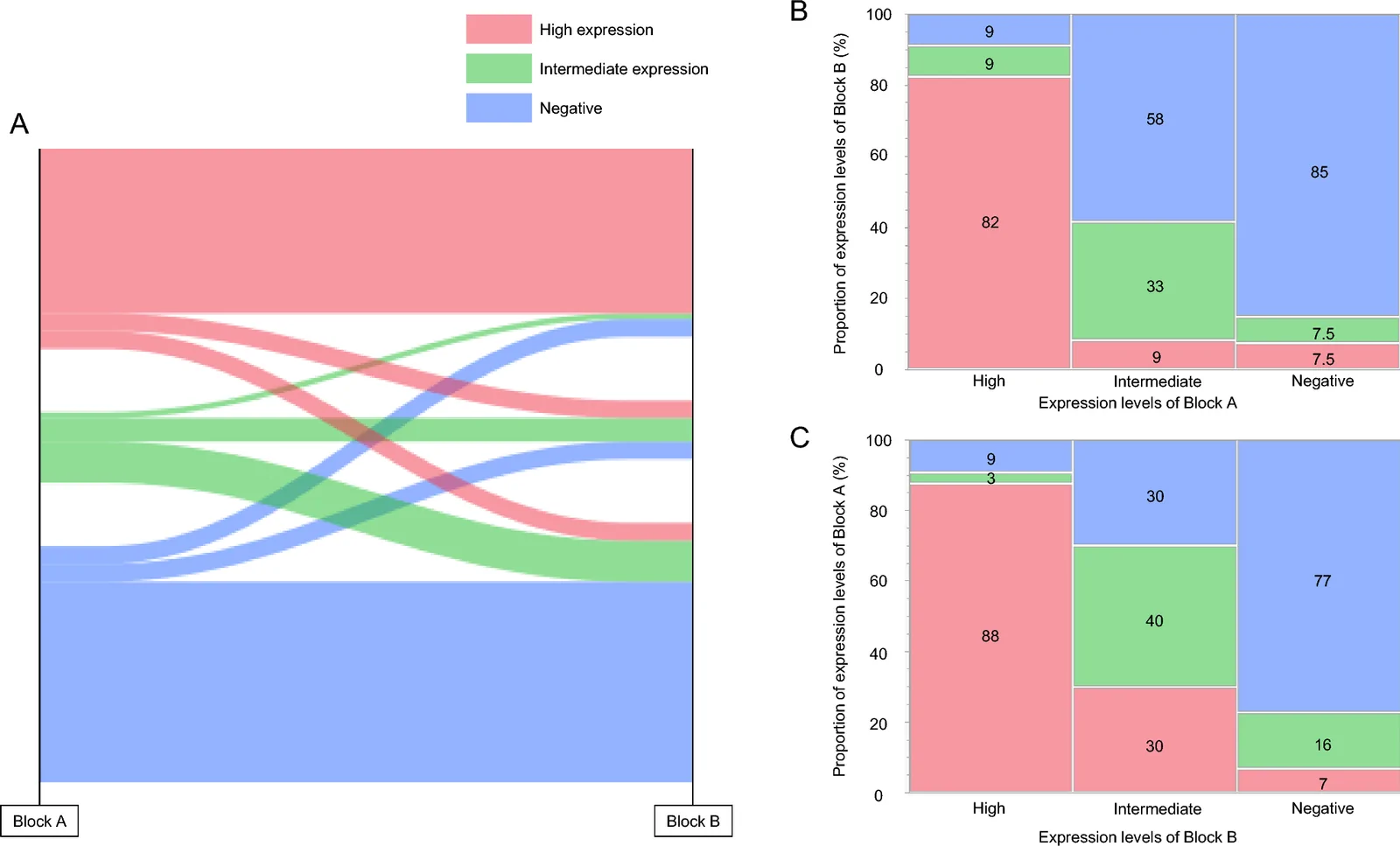
Concordance of CLDN18.2 expression levels between block A and B. Sankey diagram shows the concordance of CLDN18.2 expression levels; high (red), intermediate (green) and negative (blue) between blocks A and B (**A**). Proportion of expression levels in blocks B according to the expression levels in blocks A (**B**). Proportion of expression levels in blocks A according to the expression levels in blocks B (**C**). High, high expression; intermediate, intermediate expression; Negative, negative expression

Expression level concordance between blocks A and B were observed in 77% of cases (66/86), with a high overall agreement (κ = 0.89, Table 2). Discordance was more frequent in cases where either block showed intermediate expression. Specifically, discordance rates in block A were 18%, 67%, and 15% for high, intermediate, and negative expression, respectively; in block B, they were 12%, 60%, and 23%, respectively (Fig. 2). The average discordance rates were 15%, 63.5%, and 19% for high, intermediate, and negative expression, respectively.

Intestinal type tumors were more frequent in the concordant group than in the discordant group (50% [33/66] vs. 30% [6/20]), whereas mixed type tumors were more frequent in the discordant group than in the concordant group (12% [8/66] vs. 25% [5/20]). Other clinicopathological features were comparable between concordant and discordant groups (Online Resource 7).

### Impact of CLDN18.2 staining patterns on expression levels and inter-block concordance

Staining patterns in each block were assessed separately. In block A, 26 cases exhibited a uniform pattern, and 10 a variable pattern. In block B, 23 cases had a uniform pattern and 13 a variable pattern. Both blocks showed no differences in histological classification between uniform and variable patterns (Online Resource 8). Staining patterns showed high concordance between the two blocks (κ = 0.81, Table 3).

**Table 3:** Summary of CLDN18.2 immunohistochemistry staining patterns in each block with CLDN18.2-positive tumors

			Block B				
			Uniform		Variable		Sum
			High expression	Intermediate expression	High expression	Intermediate expression	
Block A	Uniform	High expression	**23**	***0***	*2*	*1*	26
		Intermediate expression	***0***	***0***	***0***	***0***	***0***
	Variable	High expression	0	***0***	**3**	**2**	5
		Intermediate expression	0	***0***	**1**	**4**	5
	Sum		23	***0***	6	7	36

Bold highlights indicate staining patterns concordant inter-block

Italic highlights indicate staining patterns discordant inter-block

Bolditalic characters indicate that there are no cases of intermediate expression with a uniform pattern

Weighted κ value = 0.81

Subsequently, the influence of staining patterns on expression levels was examined. In high expression cases, the uniform pattern was observed in 84% (26/31) of block A and 79% (23/29) of block B, averaging 81.5%, whereas the variable pattern was observed in 16% (5/31) of block A and 21% (6/29) of block B, averaging 18.5%. In contrast, all intermediate expression tumors showed a variable pattern (100%, Table 3).

Inter-block concordance in the high expression group differed by staining pattern. When block A exhibited a uniform pattern, 96% (25/26) of cases had high expression in block B, compared with 60% (3/5) for a variable pattern (Online Resource 9A). Similarly, when block B showed a uniform or variable pattern, the probabilities of high expression in block A were 100% (23/23) and 83% (5/6), respectively (Online Resource 9B). Mean inter-block concordance was 98.0% for uniform and 71.5% for variable patterns.

### Survival analysis according to CLDN18.2 heterogeneity

The relationship between CLDN18.2 expression heterogeneity and prognosis was assessed by comparing survival outcomes between stage I–III cases with concordant versus discordant CLDN18.2 expression across tumor blocks (Fig. 1, see Online Resource 10 for patient characteristics). Intestinal type tumors were more frequent in the concordant group (53% [32/61] vs. 19% [3/16]), and tumors originating from the upper stomach were absent in the discordant group (13% [8/61] vs. 0% [0/16]). The CLDN18.2 discordant group tended to have a more favorable prognosis than the concordant group (median RFS: not reached [95% CI 30.9–NA] vs. not reached [95% CI 26.1–NA], *p* = 0.20; median OS: 85.7 months [95% CI 46.0–NA] vs. not reached [95% CI NA–NA], *p* = 0.02, Online Resource 11A; Online Resource 11B).

Prognostic impact of staining patterns was further evaluated among stage I–III cases with CLDN18.2-positive tumors in blocks A and B (Fig. 1, see Online Resource 12 for patient characteristics). The variable group was characterized by the absence of tumors in the upper location (19% [4/21] vs. 0% [0/8]) and had greater lymph node involvement (52% [11/21] vs. 62% [5/8]). Survival outcomes were comparable between tumors with uniform and variable staining patterns (median RFS, not reached [95% CI 30.9–NA] vs. not reached [95% CI 16.2–NA], *p* = 0.70; median OS, not reached [95% CI 48.0–NA] vs. not reached [95% CI 44.3–NA], *p* = 0.80; Online Resource 11C; Online Resource 11D).

## Discussion

This study assessed intratumoral heterogeneity in CLDN18.2 expression using two FFPE blocks from resected GC specimens, stratified as high (≥ 75%), intermediate (≥ 40% to < 75%), and negative (< 40%). A prior study reported high concordance rates (> 90%) for high (≥ 75%) CLDN18.2 expression within primary tumors [18], which is the threshold for zolbetuximab eligibility. However, data on intratumoral heterogeneity, particularly inter-block concordance, are limited for tumors with intermediate expression. As emerging CLDN18.2-targeted therapies adopt lower thresholds (e.g., ≥ 40%) [8–10], understanding expression heterogeneity in this range is increasingly important.

Overall concordance in CLDN18.2 expression levels was relatively high; however, discordance was more frequent in intermediate expression tumors than in high and or negative expression tumors. Similar observations have been reported for HER2 staining [28], where equivocal cases show greater variability in staining across different tumor blocks than strongly positive ones. Our findings highlight the need for careful evaluation of CLDN18.2 expression in tumors with intermediate expression, as greater heterogeneity may affect diagnostic reproducibility and treatment stratification in future CLDN18.2-targeted strategies. In daily practice, especially for patients with unresectable or metastatic disease, CLDN18.2 expression is typically assessed using biopsy samples. Previous studies have recommended the evaluation of multiple biopsy samples, generally four to six, to account for intratumoral heterogeneity when applying a high threshold for CLDN18.2 positivity, such as ≥ 75% [19, 29]. Given the greater heterogeneity observed in tumors with intermediate expression, the use of multiple biopsy samples may be particularly important when lower thresholds are used.

Clinicopathologic features were largely comparable between concordant and discordant groups, except for histology. Intestinal type tumors were more frequent in the concordant group, whereas mixed type tumors were more common in the discordant group. This pattern aligns with prior findings of higher mixed type prevalence histology in the HER2-inter-block discordant group than in the concordant group [30]. These results suggest that CLDN18.2 expression may be associated with intratumoral heterogeneity and the underlying histological background.

Staining patterns were largely consistent, with a high level of agreement. Uniform patterns were predominantly observed in high expression tumors, whereas all intermediate expression tumors exhibited variable staining. A previous study reported an association between CLDN18.2 expression levels and staining patterns with greater heterogeneity observed in tumors showing variable rather than those with uniform staining patterns [31]; however, their assessment was based on comparisons between biopsy and surgical specimens. In contrast, we evaluated intratumoral heterogeneity using two spatially distinct tumor blocks from the same tumor, which has not, to our knowledge, been previously reported. Our findings further confirm that variable staining patterns are associated with greater intratumoral heterogeneity than uniform patterns.

Survival analysis showed that discordant expression cases tended to have more favorable survival than concordant cases. In addition, survival outcomes did not significantly differ between uniform and variable staining patterns. These results should be interpreted with caution because each group included clinicopathological factors associated with both favorable and unfavorable prognosis. Specifically, the discordant group contained fewer intestinal-type tumors, a subtype generally associated with a favorable prognosis, which would be expected to predict a less favorable outcome. Conversely, it comprised more middle-lower gastric tumors, which are also associated with a favorable prognosis and would therefore predict a better outcome [32, 33]. These clinicopathological factors thus exerted opposing influences on prognosis within the group. The variable group had more frequent lymph node involvement, which is generally associated with a poor prognosis; however, the absence of upper gastric tumors in this group may have been associated with a more favorable prognosis [34, 35]. These background imbalances likely act as confounding factors. As previously reported, CLDN18.2 is not an established prognostic biomarker [29, 36–38]. Therefore, the more favorable overall survival observed in the discordant group is most plausibly attributable to these imbalances in background prognostic factors rather than to CLDN18.2 heterogeneity itself. The present survival analysis should accordingly be considered exploratory, and further studies in more homogeneous cohorts, particularly those treated with CLDN18.2-targeted therapies, are needed to clarify the clinical relevance of CLDN18.2 heterogeneity.

This study had several limitations. First, the assessment of CLDN18.2 expression was susceptible to interobserver variability. Although we used established scoring criteria, applying automated or artificial intelligence-assisted quantification methods in future studies could improve objectivity [39]. Second, the sample size was limited, necessitating larger cohorts and multi-institutional validation to generalize findings. Third, although the focus was on spatial heterogeneity, we did not evaluate its relationship with response to CLDN18.2-targeted therapies. As no patients in this cohort received CLDN18.2-targeted therapies, direct clinical impact of CLDN18.2 heterogeneity could not be assessed. Fourth, as our cohort mostly included resectable cases, the findings may not fully represent biomarker heterogeneity and clinical characteristics observed in unresectable or metastatic disease.

In summary, our study highlights the presence of substantial intratumoral heterogeneity in CLDN18.2 expression among GC specimens, particularly in intermediate expression tumors. Although overall inter-block concordance was high, discordance was markedly higher in intermediate expression cases. Staining patterns also contributed to the intratumoral heterogeneity of CLDN18.2 expression. Although CLDN18.2 heterogeneity was not clearly associated with survival outcomes, it may pose challenges for diagnostic consistency and treatment stratification, particularly as new CLDN18.2-targeted agents adopt lower positivity thresholds. Further studies using larger, multi-institutional cohorts with standardized, reproducible assessment methods are warranted to ensure accurate patient selection.

## Supplementary Information

Below is the link to the electronic supplementary material.

Supplementary file1 (PDF 7172 KB)

Supplementary file2 (PDF 910 KB)

Supplementary file3 (PDF 62 KB)

Supplementary file4 (PDF 52 KB)

Supplementary file5 (PDF 84 KB)

Supplementary file6 (PDF 84 KB)

Supplementary file7 (PDF 80 KB)

Supplementary file8 (PDF 58 KB)

Supplementary file9 (PDF 102 KB)

Supplementary file10 (PDF 76 KB)

Supplementary file11 (PDF 354 KB)

Supplementary file12 (PDF 75 KB)

## Data Availability

The data that support the findings of this study are available upon reasonable request. Requests to access these datasets should be directed to Yusuke Kawanaka, 1954c0@med.kindai.ac.jp.

## Abbreviations

GC: Gastric cancer
HER2: Human epidermal growth factor receptor type 2
CLDN18.2: Claudin-18 isoform 2
ICI: Immune checkpoint inhibitor
IHC: Immunohistochemistry
FFPE: Formalin-fixed paraffin-embedded
RFS: Recurrence-free survival
OS: Overall survival
