# Supplementary material for: Intratumoral heterogeneity of intermediate CLDN18.2 expression in gastric cancer
Source: Int J Clin Oncol. 2026 Jul 7;31(9):2033–42. doi: 10.1007/s10147-026-03118-8 (PMC13506579; doi:10.1007/s10147-026-03118-8)
Supplement: Supplementary file 1 [file 10147_2026_3118_MOESM10_ESM.pdf]

Online Resource 10

Patient characteristics for survival analysis according to CLDN18.2 expression levels concordance or discordance inter-block

**Characteristic**	**Concordant n = 61**	**Discordant n = 16**
Age, years		
Median (range)	77 (39-90)	73.5 (36-94)
Sex, n (%)		
Male	42 (69)	13 (81)
Female	19 (31)	3 (19)
Location, n (%)		
Upper	8 (13)	0 (0)
Middle	17 (28)	5 (31)
Lower	36 (59)	11 (69)
TNM staging, n (%)		
I	14 (23)	2 (13)
II	28 (46)	8 (50)
III	19 (31)	6 (37)
T stage, n (%)		
2	23 (38)	4 (25)
3	27 (44)	8 (50)
4	11 (18)	4 (25)
N stage, n (%)		
0	27 (44)	6 (37)
1	11 (18)	3 (19)
2	11 (18)	2 (13)
3	12 (20)	5 (31)
Histology, n (%)		
Intestinal	32 (53)	3 (19)

Diffuse	17 (28)	5 (31)
Mixed	7 (11)	5 (31)
Indeterminate	5 (8)	3 (19)
Complete resection, n (%)		
R0/1	61 (100)	16 (100)
R2	0 (0)	0 (20)
Adjuvant therapy *[1]		
Yes, Regimen, n (%)	28 (60)	7 (50)
TS-1	21 (46)	7 (50)
XELOX	2 (4)	0 (0)
SOX	3 (6)	0 (0)
DS	2 (4)	0 (0)
No, n (%)	19 (40)	7 (50)

CLDN18.2, claudin-18 isoform 2; TS-1, tegafur, gimestat and otastat potassium combined drug; XELOX, combination of capecitabine and oxaliplatin; SOX, combination of tegafur, gimestat and otastat potassium combined drug and oxaliplatin; DS, combination of cisplatin and tegafur, gimestat and otastat potassium combined drug

*[1] Patients with pathological stage II or III gastric cancer were included.
