# Supplementary material for: Intratumoral heterogeneity of intermediate CLDN18.2 expression in gastric cancer
Source: Int J Clin Oncol. 2026 Jul 7;31(9):2033–42. doi: 10.1007/s10147-026-03118-8 (PMC13506579; doi:10.1007/s10147-026-03118-8)
Supplement: Supplementary file 2 [file 10147_2026_3118_MOESM11_ESM.pdf]

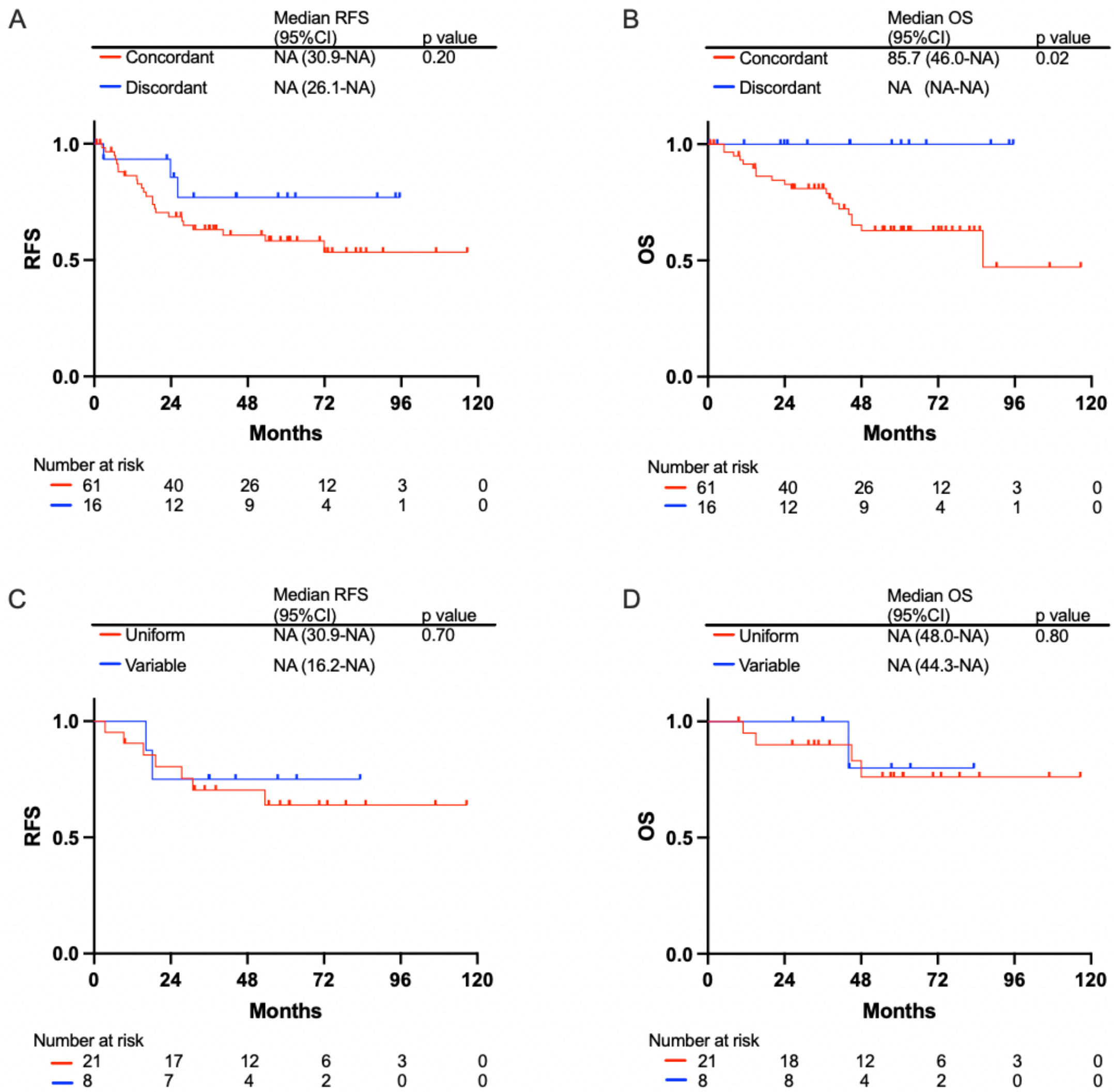

Online Resource 11

Kaplan-Meier analysis of RFS and OS based on CLDN18.2 status

RFS and OS according to CLDN18.2 expression levels concordant or discordant inter-blocks (A, B). RFS and OS according to CLDN18.2 staining patterns (C, D).

regression-free survival, RFS; overall survival, OS; CLDN18.2, claudin-18 isoform 2; NA, not applicable
