# Supplementary material for: Intratumoral heterogeneity of intermediate CLDN18.2 expression in gastric cancer
Source: Int J Clin Oncol. 2026 Jul 7;31(9):2033–42. doi: 10.1007/s10147-026-03118-8 (PMC13506579; doi:10.1007/s10147-026-03118-8)
Supplement: Supplementary file 3 [file 10147_2026_3118_MOESM12_ESM.pdf]

Online Resource 12

Patient characteristics for survival analysis according to CLDN18.2 staining pattern uniform or variable

**Characteristic**	**Uniform n = 21**	**Variable n = 8**
Age, years		
Median (range)	73 (39-85)	73 (57-87)
Sex, n (%)		
Male	12 (57)	7 (88)
Female	9 (43)	1 (12)
Location, n (%)		
Upper	4 (19)	0 (0)
Middle	6 (28)	5 (63)
Lower	11 (52)	3 (37)
TNM staging, n (%)		
I	5 (24)	1 (13)
II	10 (48)	5 (63)
III	6 (28)	2 (22)
T stage, n (%)		
2	8 (38)	3 (38)
3	9 (43)	3 (38)
4	4 (19)	2 (24)
N stage, n (%)		
0	10 (48)	3 (38)
1	4 (19)	3 (38)
2	5 (24)	1 (12)
3	2 (9)	1 (12)
Histology, n (%)		
Intestinal	11 (52)	4 (50)
Diffuse	6 (29)	2 (25)
Mixed	4 (19)	2 (25)
Indeterminate	0 (0)	0 (0)
Complete resection, n (%)		
R0/1	21 (100)	8 (100)
R2	0 (0)	0 (0)

Adjuvant therapy *[1]		
Yes, Regimen, n (%)	10 (63)	5 (71)
TS-1	7 (44)	5 (71)
XELOX	0 (0)	0 (0)
SOX	1 (6)	0 (0)
DS	2 (13)	0 (0)
No, n (%)	6 (37)	2 (29)

CLDN18.2, claudin-18 isoform 2; TS-1, tegafur, gimestat and otastat potassium combined drug; XELOX, combination of capecitabine and oxaliplatin; SOX, combination of tegafur, gimestat and otastat potassium combined drug and oxaliplatin; DS, combination of cisplatin and tegafur, gimestat and otastat potassium combined drug

*[1] Patients with pathological stage II or III gastric cancer were included.
