# Supplementary material for: Intratumoral heterogeneity of intermediate CLDN18.2 expression in gastric cancer
Source: Int J Clin Oncol. 2026 Jul 7;31(9):2033–42. doi: 10.1007/s10147-026-03118-8 (PMC13506579; doi:10.1007/s10147-026-03118-8)
Supplement: Supplementary file 4 [file 10147_2026_3118_MOESM1_ESM.pdf]

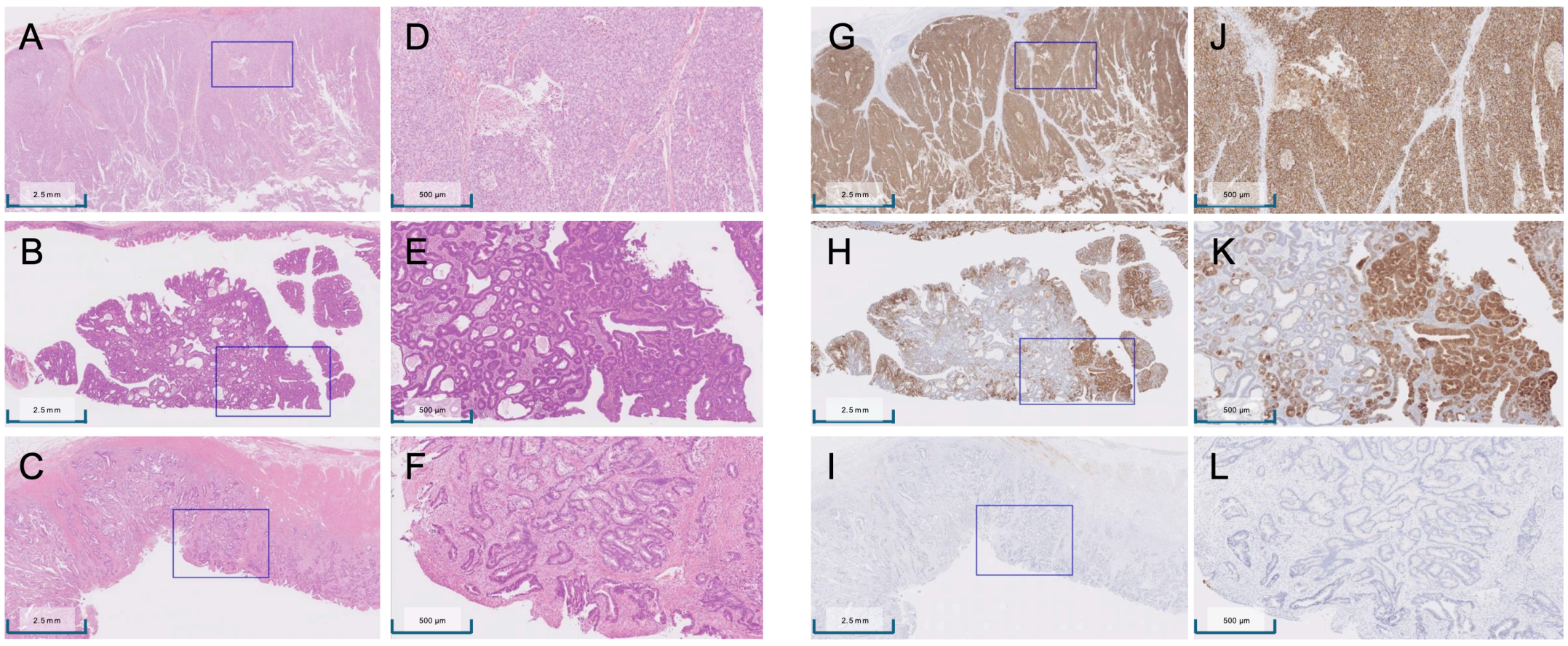

Online Resource 1

Representative images of CLDN18.2 IHC expression levels of gastric cancers

Left column shows the H&E staining of resected gastric cancer with low-magnification (A-C) and high-magnification (D-F). Right column shows corresponding CLDN18.2 IHC staining with low-magnification (G-I) and high-magnification (J-L).

Case with high expression of CLDN18.2 IHC (G, J). Case with intermediate expression of CLDN18.2 IHC (H, K). Case with negative of CLDN18.2 IHC (I, L).

CLDN18.2, claudin-18 isoform 2; IHC, immunohistochemistry; H&E, hematoxylin-eosin.
