# Supplementary material for: Intratumoral heterogeneity of intermediate CLDN18.2 expression in gastric cancer
Source: Int J Clin Oncol. 2026 Jul 7;31(9):2033–42. doi: 10.1007/s10147-026-03118-8 (PMC13506579; doi:10.1007/s10147-026-03118-8)
Supplement: Supplementary file 5 [file 10147_2026_3118_MOESM2_ESM.pdf]

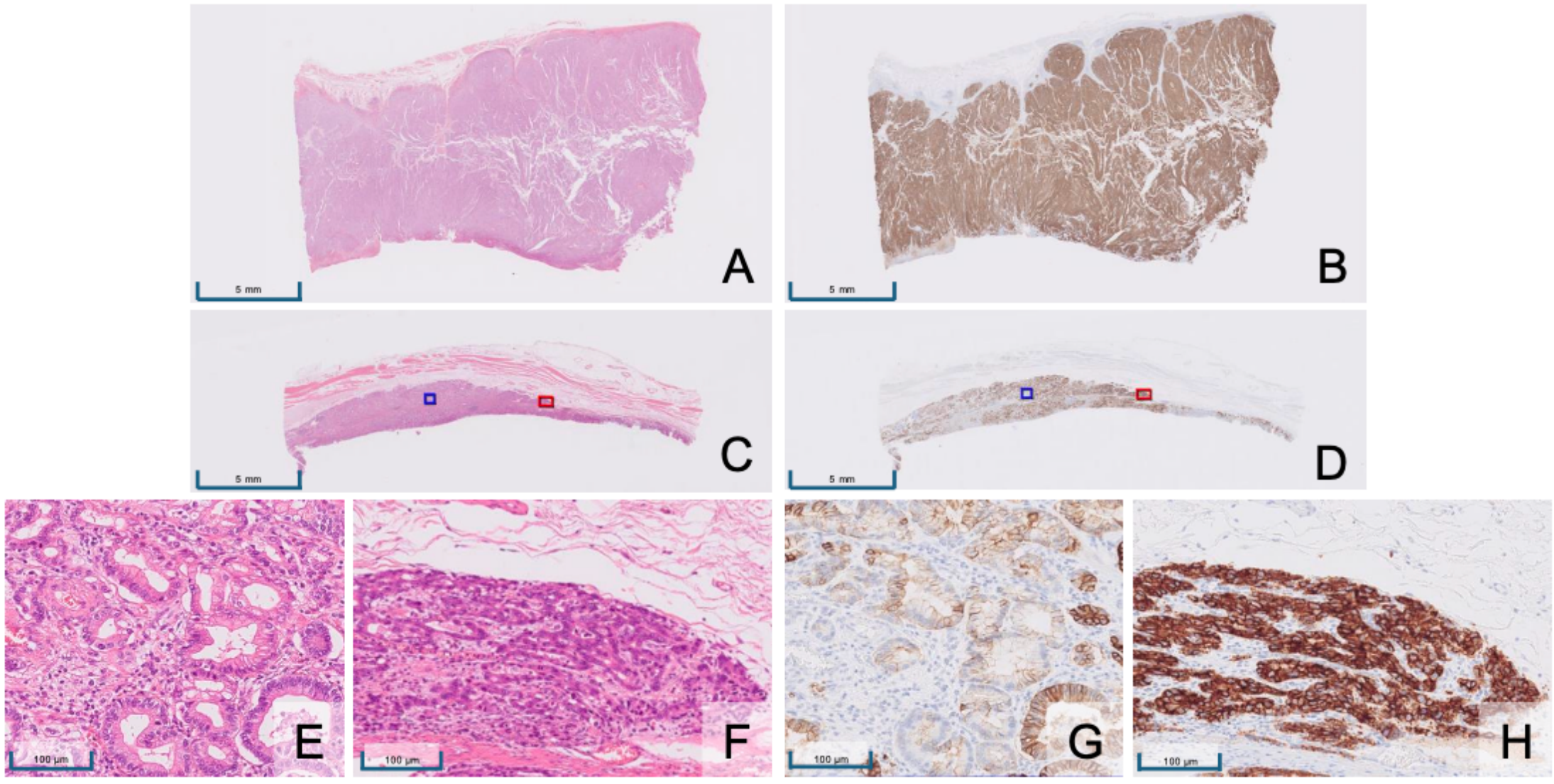

Online Resource 2

Representative images of CLDN18.2 IHC staining patterns in gastric cancers

Low-magnification H&E staining (A) and corresponding CLDN18.2 IHC (B) of a case with a uniform staining pattern. Low-magnification H&E staining (C) and corresponding CLDN18.2 IHC (D) of a case with a variable staining pattern, showing regional variation in staining intensity and distribution. (E-H) show high-magnification views of boxed regions in panels (C) and (D). Area corresponding to the blue box, showing weak/absent intensity of CLDN18.2 staining (E, G). Area corresponding to the red box, showing strong intensity of CLDN18.2 staining (F, H).

CLDN18.2, claudin-18 isoform 2; H&E, hematoxylin-eosin; IHC, immunohistochemistry.
