# Supplementary material for: Intratumoral heterogeneity of intermediate CLDN18.2 expression in gastric cancer
Source: Int J Clin Oncol. 2026 Jul 7;31(9):2033–42. doi: 10.1007/s10147-026-03118-8 (PMC13506579; doi:10.1007/s10147-026-03118-8)
Supplement: Supplementary file 6 [file 10147_2026_3118_MOESM3_ESM.pdf]

Online Resource 3

Interobserver agreement among two observers in evaluating CLDN18.2 expression levels in block A and block B

**Block A**	**Agreement**		
	**Type**	**n/N**	**%**
Between-observer	AHPA	33/34	97
	AIPA	5/12	42
	ANA	39/40	98
	OPA	77/86	90

Average High Positive Agreement (AHPA), Average Intermediate Positive Agreement (AIPA), Average Negative Agreement (ANA), Overall Percent Agreement (OPA)

Weighted κ value = 0.97

**Block B**	**Agreement**		
	**Type**	**n/N**	**%**
Between-observer	AHPA	31/32	97
	AIPA	6/10	60
	ANA	43/44	98
	OPA	77/86	93

Average High Positive Agreement (AHPA), Average Intermediate Positive Agreement (AIPA), Average Negative Agreement (ANA), Overall Percent Agreement (OPA)

Weighted κ value = 0.98
