# Supplementary material for: Intratumoral heterogeneity of intermediate CLDN18.2 expression in gastric cancer
Source: Int J Clin Oncol. 2026 Jul 7;31(9):2033–42. doi: 10.1007/s10147-026-03118-8 (PMC13506579; doi:10.1007/s10147-026-03118-8)
Supplement: Supplementary file 7 [file 10147_2026_3118_MOESM4_ESM.pdf]

Online Resource 4

CLDN18.2 expression frequency by storage period, shown as n (%) for blocks A and B.

Duration	Block	High	Intermediate	Negative
2015-2017	A	21 (42%)	7 (14%)	22 (44%)
	B	19 (38%)	5 (10%)	26 (52%)
2018-2021	A	13 (36%)	5 (14%)	18 (50%)
	B	12 (33%)	5 (14%)	19 (53%)
