# Supplementary material for: Intratumoral heterogeneity of intermediate CLDN18.2 expression in gastric cancer
Source: Int J Clin Oncol. 2026 Jul 7;31(9):2033–42. doi: 10.1007/s10147-026-03118-8 (PMC13506579; doi:10.1007/s10147-026-03118-8)
Supplement: Supplementary file 8 [file 10147_2026_3118_MOESM5_ESM.pdf]

Online Resource 5

Patient characteristics by CLDN18.2 expression levels in block A

**Characteristic**	**High expression n = 34**	**Intermediate expression n = 12**	**Negative n = 40**
Age, years			
Median (range)	74.5 (39-89)	72 (36-87)	78 (50-94)
Sex, n (%)			
Male	23 (68)	10 (83)	31 (77)
Female	11(32)	2 (17)	9 (23)
Location, n (%)			
Upper	5 (15)	1 (8)	4 (10)
Middle	12 (35)	6 (50)	8 (20)
Lower	17 (50)	5 (42)	28 (70)
TNM staging, n (%)			
I	6 (18)	2 (17)	8 (20)
II	14 (41)	6 (50)	16 (40)
III	9 (26)	2 (17)	14 (35)
IV	5 (15)	2 (17)	2 (5)
T stage, n (%)			
2	10 (29)	5 (42)	13 (33)
3	16 (47)	4 (33)	19 (47)
4	8 (24)	3 (25)	8 (20)
N stage, n (%)			
0	12 (35)	5 (42)	16 (40)
1	9 (26)	4 (33)	5 (13)
2	7 (21)	1 (8)	6 (15)
3	6 (18)	2 (17)	13 (32)
Histology, n (%)			
Intestinal	19 (55)	1 (8)	19 (48)
Diffuse	8 (24)	5 (42)	10 (25)
Mixed	5 (15)	3 (25)	5 (12)
Indeterminate	2 (6)	3 (25)	6 (15)
Heavy alcohol consumption, n (%) *[1]			
Yes	1 (3)	0 (0)	1 (3)
No	9 (26)	6 (50)	7 (17)

Unknown	24 (71)	6 (50)	32 (80)
Smoking, n (%)			
Yes	4 (12)	4 (33)	2 (5)
No	5 (15)	2 (17)	5 (13)
Unknown	25 (74)	6 (50)	33 (83)
HER2 expression, n (%)			
Positive	4 (12)	0 (0)	5 (13)
Negative	18 (53)	8 (67)	24 (60)
Unknown	12 (35)	4 (33)	11 (28)
Complete resection, n (%)			
R0/1	29 (85)	10 (83)	38 (95)
R2	5 (15)	2 (17)	2 (5)
Adjuvant therapy *[2]			
Yes, Regimen, n (%)	15 (65)	3 (37)	17 (56)
TS-1	12 (52)	3 (37)	13 (43)
XELOX	0 (0)	0 (0)	2 (7)
SOX	1 (4)	0 (0)	2 (7)
DS	2 (9)	0 (0)	0 (0)
No, n (%)	8 (35)	5 (63)	13 (44)
Recurrence *[3]			
Yes, Sites, n (%)	12 (41)	1 (10)	14 (36)
Peritoneum	5 (42)	1 (100)	7 (50)
Liver	2 (17)	0 (0)	4 (29)
Lymph node	4 (33)	1 (100)	6 (43)
Lung	1 (8)	0 (0)	2 (14)
Ascites	5 (42)	0 (0)	8 (57)
No, n (%)	17 (59)	9 (90)	24 (64)

CLDN18.2, claudin-18 isoform 2; HER2, human epidermal growth factor receptor 2; TS-1, tegafur, gimestat and otastat potassium combined drug; XELOX, combination of capecitabine and oxaliplatin; SOX, combination of tegafur, gimestat and otastat potassium combined drug and oxaliplatin; DS, combination of cisplatin and tegafur, gimestat and otastat potassium combined drug

*[1]Heavy alcohol consumption was defined as consuming ≥40 grams of pure alcohol per day.

*[2] Patients with pathological stage II or III gastric cancer were included.

*[3] Patients with pathological stage I-III gastric cancer were included.
