# Supplementary material for: Intratumoral heterogeneity of intermediate CLDN18.2 expression in gastric cancer
Source: Int J Clin Oncol. 2026 Jul 7;31(9):2033–42. doi: 10.1007/s10147-026-03118-8 (PMC13506579; doi:10.1007/s10147-026-03118-8)
Supplement: Supplementary file 9 [file 10147_2026_3118_MOESM6_ESM.pdf]

Online Resource 6

Patient characteristics by CLDN18.2 expression levels in block B

**Characteristic**	**High expression n = 32**	**Intermediate expression n = 10**	**Negative n = 44**
Age, years			
Median (range)	75 (39-87)	74.5 (61-94)	77.5 (36-90)
Sex, n (%)			
Male	22 (69)	8 (80)	34 (77)
Female	10 (31)	2 (20)	10 (23)
Location, n (%)			
Upper	5 (16)	1 (10)	4 (9)
Middle	10 (31)	4 (40)	12 (27)
Lower	17 (53)	5 (50)	28 (64)
TNM staging, n (%)			
I	5 (16)	1 (10)	10 (23)
II	15 (47)	4 (40)	17 (39)
III	8 (25)	2 (20)	15 (34)
IV	4 (13)	3 (30)	2 (5)
T stage, n (%)			
2	10 (31)	3 (30)	15 (34)
3	14 (44)	5 (50)	20 (45)
4	8 (25)	2 (20)	9 (20)
N stage, n (%)			
0	11 (35)	3 (30)	19 (43)
1	8 (25)	3 (30)	7 (16)
2	7 (22)	1 (10)	6 (14)
3	6 (19)	3 (30)	12 (27)
Histology, n (%)			
Intestinal	15 (47)	2 (20)	21 (48)
Diffuse	10 (31)	5 (50)	13 (29)
Mixed	4 (13)	2 (20)	3 (7)
Indeterminate	3 (9)	1 (10)	7 (16)
Heavy alcohol consumption, n (%) *[1]			
Yes	1 (3)	0 (0)	1 (2)
No	8 (25)	4 (40)	10 (23)

Unknown	23 (72)	6 (60)	33 (75)
Smoking, n (%)			
Yes	6 (19)	1 (10)	6 (14)
No	2 (6)	3 (30)	4 (9)
Unknown	24 (75)	6 (60)	34 (77)
HER2 expression, n (%)			
Positive	3 (9)	1 (10)	5 (11)
Negative	18 (56)	3 (30)	29 (66)
Unknown	11 (34)	6 (60)	10 (23)
Complete resection, n (%)			
R0/1	28 (88)	7 (70)	42 (95)
R2	4 (12)	3 (30)	2 (5)
Adjuvant therapy *2			
Yes, Regimen, n (%)	15 (65)	3 (50)	17 (53)
TS-1	12 (52)	3 (50)	13 (41)
XELOX	0 (0)	0 (0)	2 (6)
SOX	1 (4)	0 (0)	2 (6)
DS	2 (9)	0 (0)	0 (0)
No, n (%)	8 (35)	3 (50)	15 (47)
Recurrence *3			
Yes, Sites, n (%)	12 (41)	0 (0)	14 (36)
Peritoneum	5 (50)	0 (0)	8 (47)
Liver	2 (20)	0 (0)	3 (18)
Lymph node	2 (20)	0 (0)	7 (41)
Lung	1 (10)	0 (0)	2 (12)
Ascites	4 (40)	0 (0)	9 (53)
No, n (%)	16 (59)	7 (100)	28 (64)

CLDN18.2, claudin-18 isoform 2; HER2, human epidermal growth factor receptor 2; TS-1, tegafur, gimestat and otastat potassium combined drug; XELOX, combination of capecitabine and oxaliplatin; SOX, combination of tegafur, gimestat and otastat potassium combined drug and oxaliplatin; DS, combination of cisplatin and tegafur, gimestat and otastat potassium combined drug

*[1]Heavy alcohol consumption was defined as consuming ≥40 grams of pure alcohol per day.

*[2] Patients with pathological stage II or III gastric cancer were included.

*[3] Patients with pathological stage I-III gastric cancer were included.
