# Supplementary material for: Intratumoral heterogeneity of intermediate CLDN18.2 expression in gastric cancer
Source: Int J Clin Oncol. 2026 Jul 7;31(9):2033–42. doi: 10.1007/s10147-026-03118-8 (PMC13506579; doi:10.1007/s10147-026-03118-8)
Supplement: Supplementary file 10 [file 10147_2026_3118_MOESM7_ESM.pdf]

Online Resource 7

Patient characteristics by CLDN18.2 expression levels concordance or discordance inter-block

Characteristic	Concordant n = 66	Discordant n = 20
Age, years		
Median (range)	77 (39-90)	75 (36-89)
Sex, n (%)		
Male	46 (70)	17 (85)
Female	20 (30)	3 (15)
Location, n (%)		
Upper	10 (15)	0 (0)
Middle	19 (29)	7 (35)
Lower	37 (56)	13 (65)
TNM staging, n (%)		
I	14 (21)	2 (10)
II	28 (42)	8 (40)
III	19 (29)	6 (30)
IV	5 (8)	4 (20)
T stage, n (%)		
2	24 (36)	4 (20)
3	30 (46)	9 (45)
4	12 (18)	7 (35)
N stage, n (%)		
0	27 (41)	6 (30)
1	13 (18)	5 (25)
2	12 (18)	2 (10)
3	14 (21)	7 (35)
Histology, n (%)		
Intestinal	33 (50)	6 (30)
Diffuse	17 (26)	6 (30)
Mixed	8 (12)	5 (25)
Indeterminate	8 (12)	3 (15)
Heavy alcohol consumption, n (%) *[1]		
Yes	2 (3)	0 (0)
No	15 (23)	7 (35)

Unknown	49 (74)	13 (65)
Smoking, n (%)		
Yes	7 (11)	4 (20)
No	8 (12)	3 (15)
Unknown	51 (77)	13 (65)
HER2 expression, n (%)		
Positive	8 (12)	1 (5)
Negative	40 (61)	10 (50)
Unknown	18 (27)	9 (45)
Complete resection, n (%)		
R0/1	61 (92)	16 (80)
R2	5 (8)	4 (20)
Adjuvant therapy *[2]		
Yes, Regimen, n (%)	28 (59)	7 (50)
TS-1	21 (45)	7 (50)
XELOX	2 (4)	0 (0)
SOX	3 (6)	0 (0)
DS	2 (4)	0 (0)
No, n (%)	19 (41)	7 (50)
Recurrence *[3]		
Yes, Sites, n (%)	24 (39)	3 (19)
Peritoneum	12 (50)	1 (25)
Liver	5 (21)	0 (0)
Lymph node	7 (29)	3 (100)
Lung	3 (13)	0 (0)
Local recurrence	4 (17)	0 (0)
Ascites	12 (50)	2 (67)
No, n (%)	37 (61)	13 (81)

CLDN18.2, claudin-18 isoform 2; HER2, human epidermal growth factor receptor 2; TS-1, tegafur, gimestat and otastat potassium combined drug; XELOX, combination of capecitabine and oxaliplatin; SOX, combination of tegafur, gimestat and otastat potassium combined drug and oxaliplatin; DS, combination of cisplatin and tegafur, gimestat and otastat potassium combined drug

*[1]Heavy alcohol consumption was defined as consuming ≥40 grams of pure alcohol per day.

*[2] Patients with pathological stage II or III gastric cancer were included.

*[3] Patients with pathological stage I-III gastric cancer were included.
