# Supplementary material for: Intratumoral heterogeneity of intermediate CLDN18.2 expression in gastric cancer
Source: Int J Clin Oncol. 2026 Jul 7;31(9):2033–42. doi: 10.1007/s10147-026-03118-8 (PMC13506579; doi:10.1007/s10147-026-03118-8)
Supplement: Supplementary file 11 [file 10147_2026_3118_MOESM8_ESM.pdf]

Online Resource 8

Comparison of histological classification according to staining patterns

Histology	Block A		Block B	
	Uniform n = 26	Variable n = 10	Uniform n = 23	Variable n = 13
Intestinal, n (%)	13 (50)	5 (50)	12 (52)	6 (46)
Diffuse, n (%)	7 (27)	2 (20)	6 (26)	3 (23)
Mixed, n (%)	4 (15)	2 (20)	4 (17)	2 (15)
Indeterminate, n (%)	2 (8)	1 (10)	1 (4)	2 (15)
