# Supplementary material for: Intratumoral heterogeneity of intermediate CLDN18.2 expression in gastric cancer
Source: Int J Clin Oncol. 2026 Jul 7;31(9):2033–42. doi: 10.1007/s10147-026-03118-8 (PMC13506579; doi:10.1007/s10147-026-03118-8)
Supplement: Supplementary file 12 [file 10147_2026_3118_MOESM9_ESM.pdf]

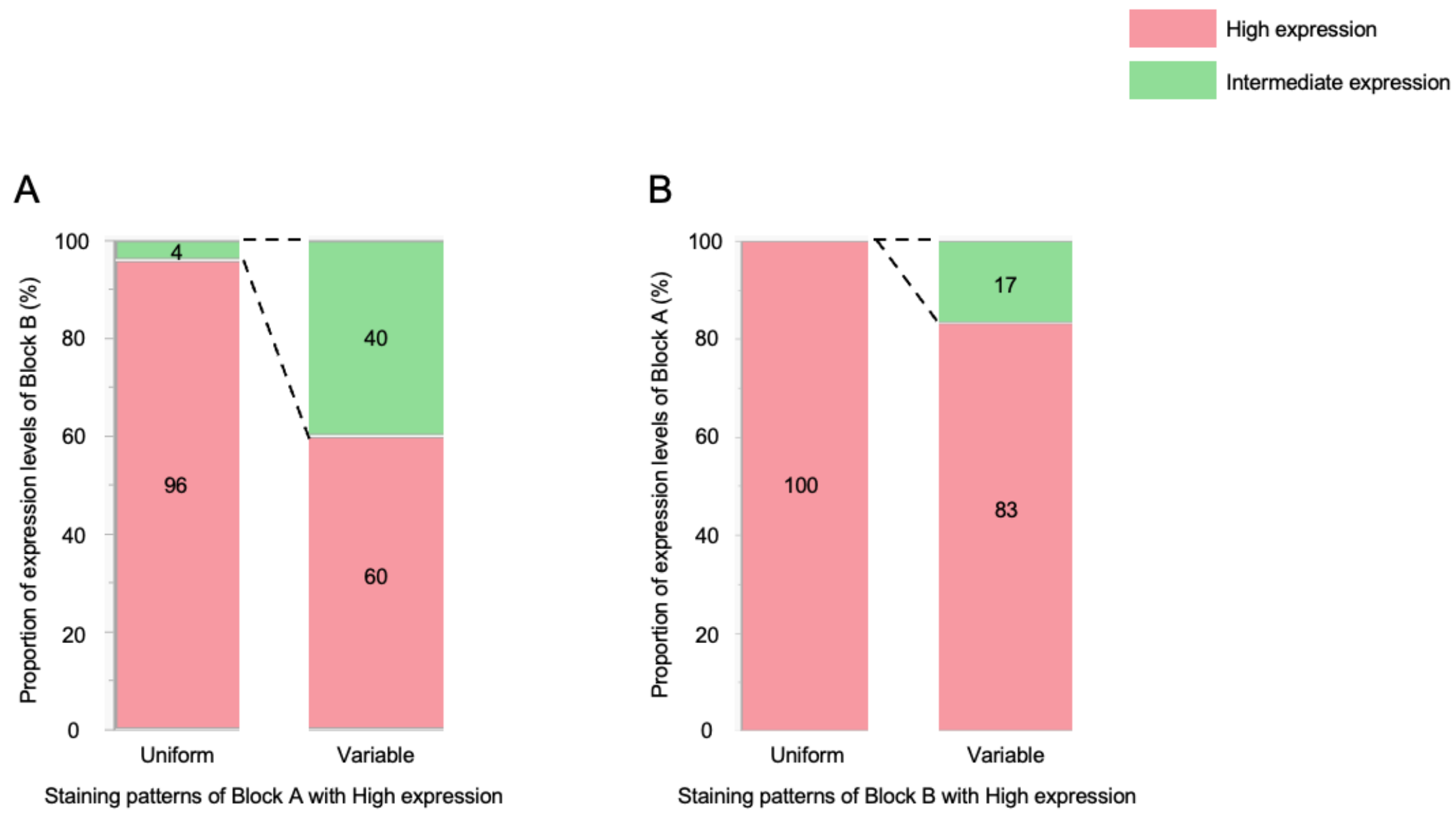

Online Resource 9

Association between staining patterns and expression levels across blocks A and blocks B

Proportion of expression levels in each block according to the staining patterns in block A or B with high expression (A, B)
